# Enabling ‘citizen voice’ in the English health and social care system: A national survey of the organizational structures, relationships and impacts of local Healthwatch in England

**DOI:** 10.1111/hex.13086

**Published:** 2020-07-07

**Authors:** Giulia Zoccatelli, Amit Desai, Graham Martin, Sally Brearley, Trevor Murrels, Glenn Robert

**Affiliations:** ^1^ Florence Nightingale Faculty of Nursing, Midwifery & Palliative Care King’s College London London UK; ^2^ The Healthcare Improvement Science Institute University of Cambridge Cambridge UK; ^3^ Faculty of Health, Social Care and Education Kingston University and St George’s University of London London UK

**Keywords:** citizen participation, community organizations, England, health and social care, Healthwatch, local government, NHS, patient and public involvement, PPI, public participation

## Abstract

**Background:**

Local Healthwatch have been operating since 2013 as ‘consumer champions’ in health and social care in England. There is little evidence about how they operate and the daily practices through which they seek to represent citizen views and influence others.

**Objective:**

To explore (a) the current organizational arrangements, relationships and impact of local Healthwatch in England, and (b) to what extent do these vary across local Healthwatch organizations.

**Design:**

An online survey of all 150 local Healthwatch in England between December 2018 and January 2019. The survey comprised 47 questions and used a combination of closed‐ and open‐response questions.

**Results:**

We received responses from 96 local Healthwatch (68% response rate). Most local Healthwatch reported that they are ‘independent’ organizations that only do Healthwatch‐related work (58.3%) and are funded through a contract (79.2%). Budget cuts have affected four‐fifths of local Healthwatch (79.3%) since 2013. Three‐quarters (74%) of local Healthwatch currently receive funding external to that provided by their local authority for their Healthwatch functions. Most Healthwatch engage with only one CCG (56.3%), one mental health trust (82.3%) and one community health trust (62.5%), though 59.4% engage with more than one hospital trust. Healthwatch respondents overwhelmingly reported impacts that were local in nature.

**Conclusions:**

Geographical and historical factors, the quality and quantity of their relationships with stakeholders, and different funding arrangements all contribute to high variability in the structure and activities of local Healthwatch and to shaping the nature of their work and impact across England.

## 1 INTRODUCTION

Enabling citizens’ voices to be heard is vital for planning the provision of publicly funded health and social care services and to ensure that the wider systems—of which such services are a part—are accountable to the public, communities and patients that they serve. In England, state‐sponsored patient and public involvement (PPI) dates to 1974, when Community Health Councils (CHCs) were established as a new model through which to represent the views of the public and advocate for local patients in each area health authority.

Since 2000, there have been three major reorganizations of the statutory system for patient and public involvement (PPI) in England.^1^, ^2^ CHCs were replaced by PPI Forums in 2002, which were themselves abolished and replaced by Local Involvement Networks (LINks) in 2008. LINks operated for only four years before they were superseded by Healthwatch, which was established as part of the Coalition Government's 2012 reform of health and social care. Each iteration of the formal PPI system in England has involved different duties, powers, funding, composition and mechanisms for accountability (see Table 1, adapted from Hogg^1^).

**TABLE 1:** Patient and Public Involvement in England, 1974 to now (adapted from Hogg, 2007:132)^1^

	1976‐2002 Community Health Councils (CHC)	2003‐2007 Patient and Public Involvement Forums (PPIF)	2008‐2013 Local Involvement Networks (LINks)	2013‐now Healthwatch
Number	185	572	151	150
Funding	Regional NHS office	Commission for Patient and Public Involvement in Health (CPPIH)	Local Authority with funding from DoHSC	Local Authority with funding from DoHSC
Cover	Locality	NHS and primary care trusts in England	Local Authority	Local Authority
Remit	NHS and public health	NHS and public health	Health and social care	Health and social care
Accountability	Unclear, but could be removed by nominating organization	Commission for Patient and Public Involvement in Health (CPPIH)	To be determined locally	Local Authority
Staff	Selected by CHC members, employed by the NHS	Employed through voluntary organizations who are contracted to support PPIF	Employed by host organizations	Employed by Healthwatch independently or through their host organizations
Statutory powers	Request information, visit NHS premises, sit as observers on health authority boards, be consulted on major changes in health care, appeal to the Secretary of State	Request information and visit NHS premises	Request information, visit NHS premises, refer health and social care matters to local council's Overview and Scrutiny Committee	Request information, visit NHS premises, sit on local statutory Health and Wellbeing Boards, signpost health and social care services, escalate issues to Healthwatch England or the Care Quality Commission

Originally conceived as a ‘consumer champion in health and care’, local Healthwatch are now ostensibly a major partner through which local government monitor the quality—and support the design—of health and social care.^3^, ^4^ Seven years since their establishment, there are 150 local Healthwatch bodies across England. Their work is supported at the national level by Healthwatch England, an independent statutory subcommittee within the Care Quality Commission (CQC), which provides local organizations with guidance and advice and draws on data collected locally to highlight national trends and issues. Commissioned by and accountable to local authorities, with funding from the Department of Health and Social Care, local Healthwatch have six statutory functions, which are outlined in Box 1. Failure to fulfil these functions may hamper proper patient and public representation in health and care planning and provision, creating a dangerous distance between local communities and the care services they need to access.

BOX 1 **The six statutory functions of local Healthwatch (readapted from https://www.healthwatch.co.uk/our‐history‐and‐functions)**

**Local healthwatch**

- Obtain the views of people about their needs and experience of local health and social care services. They make these views known to those involved in the commissioning and scrutiny of care services, like Clinical Commissioning Groups (CCGs), local authorities, and hospital trusts.
- Make reports and make recommendations about how those services could or should be improved.
- Promote and support the involvement of people in the monitoring, commissioning and provision of local health and social care services.
- Provide information and advice to the public about accessing health and social care services and the options available to them.
- Make the views and experiences of people known to Healthwatch England, supporting its role as national champion.
- Make recommendations to Healthwatch England to advise the Care Quality Commission (CQC) to carry out special reviews or investigations into areas of concern.

Local Healthwatch are differentiated from previous PPI systems principally by the above‐mentioned legally mandated functions as well as a statutory seat on local Health and Wellbeing Boards. These latter were themselves a key plank of the 2012 reforms for integrating health and social care and ensuring the inclusion of a wide range of local stakeholders in the planning of health care, social care and public health.^5^, ^6^ Healthwatch's membership of Health and Wellbeing Boards was intended to give local Healthwatch a more extensive role in the decision‐making mechanisms through which health and social care services are commissioned and provided locally. In a further major change to the health and care policy landscape since the 2012 reforms, Sustainability and Transformation Partnerships (STP) or Integrated Care Systems (ICS) are being developed throughout England; STPs and ICSs are currently emerging as key players in regional health commissioning and provision. Healthwatch is expected to be actively involved in their development, despite reports suggesting this has not always been the case to date (7 p. 37, 8 pp. 31‐32, 9).

While all Healthwatch are required to be social enterprises and are expected to involve volunteers in their activities and governance structures, there is no nationally mandated model through which a Healthwatch is required to operate. Such flexibility in terms of organizational arrangements has resulted in various models being employed. For instance, Healthwatch organizations can be registered as charities, community interest companies or private limited companies. Some may function as independent organizations which only do Healthwatch work, whereas others may be part of larger organizations which also do work unrelated to Healthwatch.

Although there have been several studies of Healthwatch's predecessors,^1^, ^10^, ^11^, ^12^, ^13^ there has been little research into how local Healthwatch bodies are organized, how they build and maintain relationships with different stakeholders, and ultimately, whether they are making a meaningful contribution as a key pillar of citizen and patient involvement in the English NHS. Mixed methods research commissioned by the Department of Health examined the initial operations of local Healthwatch 18 to 21 months since their launch and noted the early variability of Healthwatch work as well as its general reliance on positive relationships with local stakeholders in order to ‘build legitimacy, influence and create impact’.^14^ The research also highlighted activities which made Healthwatch effective in its early days and proposed recommendations for change (ibid).

More recent qualitative research on local Healthwatch in one English region has pointed to a lack of clarity of Healthwatch's role in the landscape of health and social care planning and provision.^2^, ^15^ The researchers identify what they term as the ‘jurisdictional misalignment’ between local Healthwatch, local authorities, Health and Wellbeing Boards and the NHS organizations with which they must work^5^, ^15^, ^16^ as a key challenge. Other tensions include competition with other third sector and PPI organizations and processes, and constrained local authority budgets from which local Healthwatch contracts are awarded, typically for two or three years at a time.^2^, ^15^

While these studies point to the challenges and tensions faced by local Healthwatch, they provide little evidence about the contexts in which Healthwatch operates today, the daily practices through which its influence is created and maintained, and how this enables or hampers the improvement of services for patients. Part of a broader study which will make both policy and practice recommendations, this paper starts to address these wider questions by mapping the key arrangements that structure the daily work of local Healthwatch. Drawing on the first independent national survey of the Healthwatch network, we address two research questions: what are the current organizational arrangements, relationships and impact of local Healthwatch in England? To what extent do these vary across local Healthwatch organizations?

## 2 METHODS

We conducted a national online survey between December 2018 and January 2019. The survey was registered on the King's College London Research Ethics Minimal Risk Register (MRA‐19/18‐8494).

All local Healthwatch in England were invited to take part in the survey. We obtained a list of 150 publicly available ‘info@’ email addresses of local Healthwatch from Healthwatch England and sent unique links to these addresses. We asked the local Healthwatch Chief Executive, Director or manager to complete it. The survey was conducted using the JISC Online Survey platform. We sent weekly reminders to potential respondents. We also reminded them through Facebook and Twitter and asked Healthwatch England to publicize the survey through their communication channels.

The survey was designed in consultation with:

- participants at the Healthwatch Annual Conference (mainly Healthwatch Chief Executives, Chairs and managers) in October 2018 where we ran a workshop to identify areas the survey should explore;
- Healthwatch England in relation to the surveys and data returns they already conduct and collate from local Healthwatch (to avoid duplication in our survey);
- our independent project Advisory Group, which comprises academic and professional members, including a representative of Healthwatch England, a local Healthwatch manager, a local councillor and chair of a Health and Wellbeing Board and two lay members;
- five former local Healthwatch Chief Executive Officers or Directors.

The final version of the survey had 47 questions and examined three facets of local Healthwatch work. The first section focused on Healthwatch organizational structure, particularly funding arrangements and staffing. The second focused on local Healthwatch engagement with key partners, location of relevant stakeholders and level of cooperation. The third explored the types and qualities of the impact achieved (or intended) by local Healthwatch. Based on suggestions made by the former local Healthwatch chief executives and directors who piloted our survey, in this third section we opted for descriptive questions about the types of impact achieved and about practical examples of successful or failed impact experienced by local Healthwatch in the past 3 years. This approach allowed us to account for a broad range of factors involved in successful/failed projects, for example project topics, their length, stakeholders involved and systemic challenges encountered.

The survey used a combination of open‐ and closed‐response questions. The questionnaire mainly comprised ‘yes/no’ responses (eg ‘Does your Healthwatch award funding [eg grant and contract] to other organizations?’) or the selection of possible answers from a drop‐down menu (eg ‘How would you describe the overall quality of co‐operation among key health and social care stakeholders in your local area?’ with respondents asked to indicate their views on a five‐point scale from ‘Excellent’ to ‘Poor’). Most closed questions in the survey included an ‘Other’ option and allowed for free‐text responses in the form of a brief description.

Open questions were limited to the last two sections of the survey. Here, we asked respondents to briefly outline two specific pieces of work they had carried out in the past three years which they regarded as (a) successful, and (b) unsuccessful. In these final sections, we used a combination of closed and open questions, requiring text responses in the form of a brief description. Open‐ended questions asked, for example, ‘what was the piece of work about?’, ‘how was the impact delivered?’, and in the case of unsuccessful projects, ‘what barriers did your Healthwatch experience in its work?’. We coded answers based on topic, duration of the project (one year or less, between more than a year and less than two, two or more years) and barriers to impact. Closed questions in these final two sections included ‘type of impact achieved or intended to be achieved’ (covering 13 options, eg ‘Improved access to care and treatment for members of our community’; participants could select more than one option), and in the case of successful projects, ‘most important stakeholders involved’ (covering 19 options, including an ‘Other’ option; participants could select up to three).

For data about numbers of staff (total and FTE) and volunteers and Healthwatch grant/contract values, we relied on data compiled by Healthwatch England in the period 2013‐2018. These data were shared with the research team in February 2019.

A copy of the survey questionnaire is included as supplementary Material to the paper.

## 3 RESULTS

We received responses from 96 local Healthwatch. This was a response rate of 68% (as eight Healthwatch responded on behalf of two or more Healthwatch which they operated as a combined organization). Nineteen of our respondents were commissioned by county councils (19.8%), 16 by London Boroughs (16.6%), 23 by metropolitan districts (24%) and 38 by unitary authorities (39.6%). Table 2 presents a breakdown of the responses we obtained based on geographical region.

**TABLE 2:** Number of respondents by region

Region	Total number of HW	Survey respondents
East*	11	9
East Midlands**	10	6
London	32	16
North East	12	6
North West**	23	12
South East*	18	15
South West*	15	11
West Midlands	14	13
Yorkshire and Humber	15	8
Total	150	96

Note

Asterisks indicate the number of HW in each region which provided one single response on behalf of two or more HW.

### 3.1 Organizational structure

#### 3.1.1 Independent or ‘hosted’?

We categorized local Healthwatch as to whether they are (a) independent, standalone organizations that only conduct Healthwatch work in one locality or (b) are part of other organizations which also carry out other work. These latter Healthwatch are referred to here as ‘hosted’. Host types vary greatly across the Healthwatch network and include.

- local community and voluntary sector support organizations which may hold several Healthwatch contracts;
- a local social enterprise (eg disability charity) which holds the local Healthwatch contract or grant alongside other activities; or
- a local Healthwatch which now holds the contract for additional Healthwatch and does no other non‐Healthwatch work.

Most Healthwatch reported being ‘independent’ (n = 56, 58.3%); 40 Healthwatch (41.7%) said they were ‘hosted’.

To investigate whether geographical size or complexity of local authority structures was associated with whether a local Healthwatch is independent or hosted, we cross‐tabulated the above categorizations by size and type of local authority in which each Healthwatch principally operate. There are four types of local authority in England which fund the work of local Healthwatch: county, unitary, metropolitan district and London borough. Of these, counties are generally larger and more complex than the other three types. This is mainly because counties have two tiers of local government, which means powers and responsibilities are split between county‐level government and district‐level local government; Healthwatch could potentially operate at both these tiers. The other three types of local authority have a single tier. We found that a larger proportion of Healthwatch in counties describe themselves as ‘independent’ (78.9%) than in unitary authorities (47.4%), metropolitan districts (56.5%) or London boroughs (62.5%). Conversely, Healthwatch in unitary local authorities tend to report a higher proportion of hosted organizations (52.6%) than those in counties (21.1%), London (37.5%) or metropolitan (43.5%) boroughs.

#### 3.1.2 Contracts or grants?

We also explored the different mechanisms by which Healthwatch are funded by their local authority. The main difference between contracts and grants is that the former must be tendered according to government (UK and EU) procurement regulation. This process requires providers of local Healthwatch services to devote considerable time and resources to the management and renewal of their contract. Grants are not subject to these formalities; applying for a continuation of funding may not be as onerous for grant‐funded Healthwatch, and the terms of a grant may be less prescriptive or exacting than a contract. On the other hand, grants are normally provided for shorter periods of time (usually a year). Shorter funding periods could have an impact on the long‐term planning ability of a local Healthwatch.

We found that the majority of local Healthwatch (n = 76, 79.2%) are currently funded through a contract, whereas a fifth are funded by a grant (n = 19, 19.8%). One respondent chose the ‘Other’ option and explained in the free‐text section that their funding mechanism was currently under review—probably moving from grant to contract. Geographical and local authority‐based variations appeared to play a role in determining the funding mechanisms for Healthwatch. For example, although contracts are the main funding mechanism across Healthwatch in England generally, the East of England is the only region in which the number of Healthwatch with grants outnumbers those with contracts. Grants make up a larger proportion of funding mechanisms than the England average in counties (n = 5, 26.3%) and unitary local authorities (n = 9, 23.7%), whereas Healthwatch in London Boroughs (n = 2, 12.5%) and metropolitan local authorities (n = 3, 13.0%) reported lower proportions of grants than the national picture. We found a much smaller proportion of hosted Healthwatch hold grants (n = 2, 5.0%) compared to those describing themselves as ‘independent’ (n = 17, 30.4%).

#### 3.1.3 External funding

Since their launch in 2013, local Healthwatch have undergone significant budget cuts. Publicly available data compiled by Healthwatch England show the value of contracts or grants was reduced in four‐fifths of all 150 Healthwatch in England (n = 121, 79.3%) between 2013 and 2018, with nine Healthwatch experiencing cuts in excess of 50% of their original budget. It is interesting to note that, in parallel, the number of Healthwatch seeking and receiving funding beyond that provided by their local authority for their Healthwatch functions is thought to have increased since 2013.^17^ In our survey, we found that 71 Healthwatch (74.0%) were receiving such funding. The two most common services provided in exchange were ‘research on patient or service user experience’ (n = 56, 77.8%) and ‘development of patient/public engagement activities’ (n = 44, 61.1%). The sources of this external funding also varied. Forty‐four (62.0%) of the 71 Healthwatch respondents who reported receiving this funding said they received it from CCGs: 42 (59.2%) from local authorities, 25 (35.2%) from NHS providers and 24 (33.8%) from Sustainability and Transformation Partnerships (STP). Funding sources varied based on local authority types and the geographical location of local Healthwatch. For example, we found that all Healthwatch respondents from London boroughs which reported receiving external funding did so from the health sector. Conversely, outside London, the main source of funding for local Healthwatch was reported to be their local authority.

#### 3.1.4 Healthwatch as award funders

Twenty‐seven (28.1%) Healthwatch awarded funding to other organizations. Examples included contracting voluntary and community organizations to gather feedback from groups of people whom the Healthwatch found hard to reach or setting up small community funding schemes which were used to engage local organizations to carry out research or engagement with specific patient groups.

#### 3.1.5 Staffing

Publicly available data compiled by Healthwatch England for the period between April 2017 and March 2018 show that for those Healthwatch responding to the survey, the median number of total employed staff was 6 (range 2‐15); median full‐time equivalent (FTE) staff was 3 (range 1‐13.5); and the median number of volunteers was 23 (range 3‐743).

Overall, volunteers were reported to contribute significantly to ‘Enter and View’ visits. These visits are statutory powers used by Healthwatch to observe and gather information from staff and users of health and social care services at sites of care (eg a GP surgery or a care home) in order to assess the quality and standard of care. Forty‐two (43.8%) Healthwatch said that these were carried out ‘mostly by volunteers with some employed staff contribution’; 29 (30.2%) said that they were ‘equally carried out by employed staff and volunteers’. Conversely, administrative and clerical work (n = 95, 99.0%), research and report writing (n = 87, 90.7%), and communications and social media (n = 92, 95.8%) were either ‘wholly carried out by employed staff’ or ‘mostly by employed staff with some volunteer contribution’.

### 3.2 Relationships

In order to build a picture of the network of Healthwatch relationships, we asked how many CCGs, hospital trusts, mental health trusts, community health trusts, GP surgeries and care homes Healthwatch respondents engaged. We found that

- 54 (56.3%) Healthwatch respondents engage with only one CCG. A small number of Healthwatch engage with five or more CCGs (n = 9, 9.4%).
- 39 (40.6%) Healthwatch respondents engage with only one hospital trust. Six (6.2%) Healthwatch engage with five or more.
- 79 (82.3%) Healthwatch respondents engage with only one mental health trust.
- 60 (62.5%) Healthwatch respondents engage with only one community health trust. 23 (24.0%) do not engage with any community health trusts.
- 40 (41.7%) Healthwatch respondents engage with more than 40 GP surgeries.
- A third of all respondents (n = 32, 33.3%) engage with more than 50 care homes. Five (5.2%) respondents engage with none.

To provide further insight into the institutional and relational complexity of Healthwatch networks, we also asked whether local Healthwatch only engaged with stakeholders within the boundaries of their local authority. Two‐fifths of all Healthwatch respondents (n = 40, 41.7%) said this was the case. However, there was variation by (a) local authority type and (b) type of health or social care organization. Healthwatch in unitary local authorities are more likely than others to engage with organizations outside the boundaries of their local authority. For example, more than two‐fifths (42.1%) of Healthwatch located in unitary local authorities engage with CCGs outside their local authority area, compared to only three (15.8%) of those Healthwatch in counties, four (17.4%) of those in metropolitan districts and three (18.8%) in London boroughs. Healthwatch in unitary authorities are also more likely than Healthwatch in other local authority types to engage hospital trusts outside their local authority area (47.4%). This compares to seven (30.4%) of those in metropolitan districts and five (26.3%) of those in counties.

#### 3.2.1 Quality of relationships

We asked how local Healthwatch rated (a) the overall quality of the cooperation among key health and social care stakeholders in their local area, and (b) their level of engagement in the development of planning frameworks for health and social care services (eg STPs and ICSs). Most Healthwatch reported both positive relationships among local stakeholders and a good level of involvement in STPs and ICSs (Table 3). However, the survey highlighted significant regional variation across the network. For instance, we found that five out of six Healthwatch respondents in the North East of England reported having no or limited involvement in STP and ICS development.

**TABLE 3:** How would you describe the overall quality of co‐operation among key health and social care stakeholders in your local area, and to what extent has your Healthwatch been involved in the development of STPs/ICSs?

Quality of co‐operation in local area		Involvement in development of STPs/ICSs	
Excellent	7 (7.3%)	A high level of involvement	14 (14.6%)
Good	54 (56.3%)	A good level of involvement	38 (39.6%)
Neither good nor bad	20 (20.8%)	Some involvement	31 (32.3%)
Limited	15 (15.6%)	Not much involvement	8 (8.3%)
Poor	0	No involvement	5 (5.2%)

More than half of Healthwatch (n = 31, 57.4%) that reported a ‘good’ overall quality of cooperation among stakeholders in their area reported either a ‘high’ or ‘good’ involvement in STPs/ICSs. In contrast, three‐fifths of Healthwatch (n = 9, 60.0%) in areas of ‘limited’ cooperation reported only ‘some’ or ‘not much’ involvement in STPs and ICSs.

### 3.3 Impact

Healthwatch overwhelmingly reported impacts that were local in nature. The most common response among the 13 options provided was ‘Improved access to care and treatment for members of our community’, selected by 73 (76.0%) Healthwatch, followed by ‘Increased levels of participation in co‐production of people who use a service’ (n = 65, 67.7%). National‐level impacts were selected by far fewer respondents: 10 (10.4%) local Healthwatch reported that they had influenced changes in national policy or specialist commissioning and eight (8.3%) had escalated an issue to Healthwatch England which was later actioned.

We explored the relationship between the number of ‘full‐time equivalent’ (FTE) staff and (a) the number of types of local impact reported by respondents, and (b) whether they reported national impact. We found that the greater the staff FTE, the greater the number of types of local impact as well as the greater the likelihood of reporting a national impact.

#### 3.3.1 Examples of successful impact

We asked respondents to identify a successful piece of work they had completed in the past three years. The responses represent a broad range of cases of perceived impact achieved by local Healthwatch, along with an indication of the type of impact, the time needed to achieve that impact, the ways in which impact was delivered, and three key stakeholders involved in each piece of work. In Box 2, we present two examples of the returns we obtained in this section of the survey.

BOX 2 **Free text examples of successful impact**

**EXAMPLE #1**

**What was the piece of work about?**

Activities in Care Homes. Study looking at the level of activities in care homes and the impact upon the well‐being of residents.

**What was the key impact you achieved?**

Other—influenced Care Home providers to develop their activity programmes to offer a more varied and stimulating programme of activities for residents.

**How long did it take to achieve this impact?**

12 months.

**How was the impact delivered (eg research presenting evidence, publicity activity etc)?**

Research, followed by a conference, social media attention.

**Which of the following local stakeholders did you involve to achieve this impact? Please select the three most important**

- Social care providers
- Media
- Local CQC inspectors

**EXAMPLE #2**

**What was the piece of work about?**

Access to eyecare—to give people a strong voice and ensure their experiences and views are considered and influence how eye care services are provided.

**What was the key impact you achieved?**

Improved access to care and treatment for members of our local community.

**How long did it take to achieve this impact?**

While the project took two years from proposal through to our final evidence‐based research report, action was quickly taken based on our recommendations.

**How was the impact delivered (eg research presenting evidence and publicity activity)?**

Evidence/ findings presented in a research report following focus groups, site visits and interviews with members of the public.

**Which of the following local stakeholders did you involve to achieve this impact? Please select the three most important**

- Other—Local Eye Health Network
- Local patient or condition‐specific groups
- Community voluntary sector organizations

The topics covered in the examples chosen by Healthwatch respondents varied, with hospital care (n = 14, 16.5%), primary care (n = 11, 12.9%), social care (n = 10, 11.8%) and disability (n = 10, 11.8%) being the most common (see Table 4).

**TABLE 4:** Examples of successful impact: topics and project duration

Topics	Project duration			Total number (%)
	≤1 y	>1 and <2 y	≥2 y	
Hospital care	10	1	3	14 (16.5)
Disability (excluding mental health)	7	3	0	10 (11.8)
Primary care: GPs, eye care, 111 (no dentistry)	6	4	1	11 (12.9)
Social care	8	2	0	10 (11.8)
General engagement activities with patients and the public	5	2	1	8 (9.4)
Mental health	4	3	1	8 (9.4)
Children and young adults: general	4	3	0	7 (8.2)
Seldom‐heard groups: other (eg homelessness, drug & alcohol abuse, prisoners)	4	1	0	5 (5.9)
Dentistry	5	0	0	5 (5.9)
Palliative care and end of life care	3	0	0	3 (3.5)
Seldom‐heard groups: Black Minority Ethic and Refugees (BMER)	0	0	1	1 (1.2)
Service user transport	1	1	0	2 (2.4)
Carers	0	1	0	1 (1.2)
Intermediate Care	1	0	0	1 (1.2)
Phlebotomy	1	0	0	1 (1.2)
Other	1	0	0	1 (1.2)
Total	58 (68.2%)	20 (23.5%)	7 (8.2%)	85 (10%)

Regarding the type of impact achieved, almost a third of Healthwatch respondents (n = 29, 30.2%) selected a project that led to an ‘improvement in the access to care and treatment for the members of their community’. Sixteen Healthwatch (16.7%) selected an initiative through which they ‘influenced new commissioning or commissioning intentions’; 12 (12.5%) chose a project that ‘produced changes to local contract specifications’ and the same number chose a project that ‘improved the quality of care’.

The most commonly reported stakeholders involved in successful projects were ‘CCG board and staff’ (n = 40, 41.7%), ‘service users or service users groups’ (n = 33, 34.4%), ‘Health and Wellbeing Board members’ (n = 26, 27.1%), ‘Community voluntary sector organizations’ (n = 25, 26.0%) and ‘Local authority Overview and Scrutiny Committee’ (n = 21, 21.9%). Conversely, ‘Governors of Trusts’ (n = 0), ‘Local MPs’ (n = 1, 1%), ‘NHS England’ (n = 2, 2.1%), ‘staff at neighbouring Healthwatch’ (n = 3, 3.1%) and ‘local STP/ICS boards’ (n = 3, 3.1%) were only selected by a limited number of respondents.

#### 3.3.2 Examples of failed impact

We asked respondents to briefly outline a piece of work they had completed in the past three years which they regarded to have been unsuccessful. We also asked to select the type of impact they wanted to achieve and to describe the main barriers to impact they faced on its delivery (Box 3).

BOX 3 **Free text examples of failed impact**

**EXAMPLE #1**

**What was the piece of work about?**

Need for residents with autism.

**What was the impact you wanted to achieve?**

Promote issues which were adopted into a strategy (locally, regionally or nationally).

**Please briefly describe the barriers to impact you experienced**

The commissioner writing the strategy was really engaged and also put us in contact with a variety of relevant departments and NHS commissioners (who actually ended up acting on our feedback and making a change on their side). However, the commissioner left, and the posts responsibilities were left vacant for some time. We are still waiting for an opportunity to discuss the findings again. A board set up to look at the strategy did discuss the report and told us it was insightful but we have not been able to look at a longer term influence.

**EXAMPLE #2**

**What was the piece of work about?**

Community Dental Services—access to procedures carried out under general anaesthesia.

**What was the impact you wanted to achieve?**

Improve access to care and treatment for members of our local community.

**Please briefly describe the barriers to impact you experienced**

We ended up in a morass of different organizations with different responsibilities. Not everything they were each telling us could be true, as they were contradictory. The commissioner (NHS England) has been helpful in some ways but defensive in others. But we have not given up. We continue to press for answers. It is over 2 years since we began work on this.

Local Healthwatch respondents chose examples of unsuccessful projects that covered a broad range of topics. The most common were primary care (n = 17, 17.7%), hospital care (n = 14, 14.6%), disability (n = 10, 10.4%) and mental health (n = 9, 9.4%). Regarding the type of impact intended to be achieved, the majority of our Healthwatch respondents (n = 45, 46.9%) selected projects that intended to ‘improve access to care and treatment’ for members of their local community.

The two most common barriers to impact identified by local Healthwatch were the ‘lack of cooperation among or by key institutional stakeholders’ (n = 36, 37.5%), and the ‘systemic complexity or lack of clarity among stakeholders about respective organizational roles, responsibilities’ (n = 27, 28.1%), which when combined were selected by almost two thirds of our survey respondents. Despite widespread concern about decreasing Healthwatch budgets, only ten (10.4%) local Healthwatch identified a ‘lack of resources’ as the main barrier to impact.

## 4 DISCUSSION

Launched in 2013 by the Coalition Government, Healthwatch is the latest in a long line of attempts to guarantee patients and communities a say in the planning and provision of local health and social care services. Contrary to its predecessor—LINks—which were always hosted by another organization, Healthwatch were given flexibility in terms of the model under which to function. Six years since the beginning of their operations, our survey explored the current organizational arrangements, relationships and impact of local Healthwatch in England and examined the extent to which these vary across the local Healthwatch network.

Our findings bring to the fore the variability in Healthwatch arrangements and highlight some interesting trends. In terms of organizational structure, while a majority of Healthwatch do indeed operate as independent social enterprises, the number of ‘hosted organizations’ is still significant, with more than two‐fifths reporting being run by a host. Types of hosts also vary greatly, ranging from small social enterprises to large organizations which hold the contracts of several Healthwatch even in geographically dispersed areas. Only Healthwatch within unitary local authorities are more likely to be hosted—rather than independent—organizations. Healthwatch in counties, conversely, report the smallest proportion of hosted organizations. One reason for this may be that Healthwatch which are hosted by another organization may struggle to operate at the larger geographical scale of a county. While economies of scale in terms of back office functions are likely to make large host organizations more competitive in the tender for a Healthwatch contract, they may be easier to realize in smaller geographical areas (unitary local authorities) rather than in larger areas (counties). Alternatively, it might be a function of the relative population density of the different local authority types. Based on data from the Office for National Statistics, none of the county councils falls into in the top 50% of local authority areas by population density (see https://www.ons.gov.uk/file?uri=/peoplepopulationandcommunity/populationandmigration/population), and it is therefore plausible that the third sector in such comparatively sparsely populated locales is less developed than in London boroughs, unitary authorities and metropolitan districts. This may mean that there are fewer potential host organizations that might bid for Healthwatch contracts in county council areas.

Survey responses also mirror the challenging financial landscape in which most local Healthwatch currently operate. As data from Healthwatch England reveal, four out of five local Healthwatch have seen their budget reduced since 2013 and cutting operational costs as well as finding alternative sources of funding have become important for securing Healthwatch's organizational sustainability. We found that over 70% of Healthwatch are now receiving external funds in addition to their core local authority budgets. These funds cover a broad range of activities, ranging from research on patients and service users’ experiences to the provision of the NHS Complaints Advocacy Service. Sources of funding also vary greatly across the network; most common are funds from health organizations and local authorities but with geographical variation. In the face of shrinking core funding, such ancillary funding may well be a vital supplement to ensure the viability of some local Healthwatch. However, given the importance attached to their role as the principal conduit for the views of patients and service users on health and social care, dependence on these extra sources of funding may bring with them challenges around autonomy.

The variety of organizational and funding arrangements mirrors the diversity in the type and complexity of relationships with key stakeholders in health and social care, like Clinical Commissioning Groups (CCGs), acute, community and mental health hospitals, GPs and care homes. While many Healthwatch engage in relatively simple networks featuring only a limited number of local stakeholders, all located within the boundaries of their local authorities, other Healthwatch are embedded in more complex networks involving large numbers of commissioners and providers of health and social care services (eg five or more CCGs, five or more hospital trusts) located both within and outside the boundaries of their local authority. The range and quality of these relationships is likely to have a significant effect on the organization of the daily work and the potential impact of Healthwatch. These issues need further investigation and will be a key element of inquiry in the second phase of our study consisting of ethnographic fieldwork over twelve months at five purposively sampled Healthwatch.

Looking more broadly at the quality of the relationship between local stakeholders and the level of involvement reported by Healthwatch in the development of key planning frameworks for health and social care services (STPs and ICSs), our findings highlighted further variation. For instance, we found that while most Healthwatch reported a high or good level of involvement in STPs and ICSs, five out of six Healthwatch respondents in the North East of England reported having no or limited involvement in their development. Historically low levels of patient and public and/or voluntary sector involvement in the running of local services were indicated as a possible reason but will require more in‐depth investigation during the ethnographic phase of the study.

The quality of collaborative relationships with a range of partners also appeared crucially implicated in the impacts described by participating Healthwatch—and in cases where impact had not been achieved. Perhaps surprisingly, resource limitations were only indicated as the most fundamental obstacle to impact by a tenth of our respondents. This suggests that strong local impacts could be achieved within resource constraints, if productive relationships with the right stakeholders were in place. Local impacts predominated over national‐level impact, reflecting the remit of local Healthwatch, but the fact that only one in 10 participating Healthwatch felt they had influenced a national‐level policy change suggests scope for further coordination of such activity, with a key role for Healthwatch England in securing wider impact.

The findings we present in this paper are part of an ongoing study which comprises three other research phases, including a twelve months long ethnographic study of five purposely sampled local Healthwatch, six sense‐making workshops with research participants and relevant local and national stakeholders and a ‘Delphi’ analysis of our findings. As such, this paper is limited in its ability to draw wide‐ranging conclusions about more nuanced aspects of local Healthwatch work, like for example, specific challenges and strategies to maximize their impact. Our approach to the investigation of local Healthwatch impact also limits the breadth of the conclusions we are able to draw in this paper. In the survey, we opted for qualitative questions about types of impact and about specific examples of impact achieved or failed by each local Healthwatch respondent. Instead, we avoided more general questions about the overall impact of each organization. This was because we regard ‘impact’ as the relative outcome of a complex array of interrelated factors, which are better suited to the in‐depth qualitative investigation we carry out in the latter phases of this study. One limitation to the usefulness of this kind of self‐reported information on impact is that we unable at this stage of research to draw conclusions as to whether particular organizational arrangements and relationship types lead to better impact among our local Healthwatch respondents.

## CONFLICT OF INTEREST

The authors declare that there is no conflict of interest.

## Supporting information

Supplementary Material

## Data Availability

The data that support the findings of this study are available on request from the corresponding author. The data are not publicly available due to privacy or ethical restrictions.

## References

[1] Hogg CNL . Patient and public involvement: what next for the NHS? Health Expect. 2007;10(2):129‐138. 17524006 10.1111/j.1369-7625.2006.00427.x PMC5060383

[2] Martin GP , Carter P . Patient and public involvement in the new NHS: choice, voice, and the pursuit of legitimacy In: Bevir M, Waring J, eds. Decentring Health Policy: Learning from British experiences in healthcare governance. London: Routledge; 2017.

[3] Department of Health . Local Healthwatch: A Strong Voice for People – The Policy Explained. London: Department of Health; 2012.

[4] England NHS . Patient and Public Participation in Commissioning Health and Care: Statutory Guidance for CCGs and NHS England. London: NHS England; 2017.

[5] Coleman A , Checkland K , Segar J , McDermott I , Harrison S , Peckham S . Joining it up? Health and wellbeing boards in English local governance: evidence from clinical commissioning groups and shadow health and wellbeing boards. Local Government Stud. 2014;40(4):560‐580.

[6] Humphries R . Health and wellbeing boards: policy and prospects. J Integr Care. 2013;21:6‐12.

[7] Alderwick H , Dunn P , McKenna H , Walsh N , Ham C . Sustainability and Transformation Plans in the NHS How are They Being Developed in Practice?. London: The King's Fund; 2016.

[8] Ham C , Alderwick H , Dunn P , McKenna H . Delivering Sustainability and Transformation Plans: From Ambitious Proposals to Credible Plans. London: The King's Fund; 2017.

[9] Martin GP , Carter P , Dent M . Major health service transformation and the public voice: conflict, challenge or complicity? J Health Serv Res Policy. 2018;23(1):28‐35. 28870096 10.1177/1355819617728530 PMC5768261

[10] Baggott R . A funny thing happened on the way to the forum? Reforming patient and public involvement in the NHS in England. Public Administration. 2005;83(3):533‐551.

[11] Martin GP . Whose health, whose care, whose say? Some comments on public involvement in new NHS commissioning arrangements. Crit Public Health. 2009;19(1):123‐132.

[12] Taylor J , Tritter JQ . Local Involvement Networks: Learning from the Early Adopter Programme. Coventry: National Centre for Involvement; 2007.

[13] Centre for Public Scrutiny . Smoothing the Way: Developing Local Healthwatch Through Lessons from Local Involvement Networks. London: CFPS; 2012.

[14] Gilburt H , Dunn P , Foot C . Local Healthwatch: Progress and Promise. London: The King's Fund; 2012:14.

[15] Carter P , Martin GP . Challenges facing healthwatch, a new consumer champion in England. Int J Health Policy Manag. 2016;5(4):259‐263. 27239869 10.15171/ijhpm.2016.07 PMC4818991

[16] Hunter DJ , Perkins N , Visram S , et al. Evaluating the Leadership role of Health and Wellbeing Boards as Drivers of Health Improvement and Integrated Care Across England. London: National Institute for Health Research.

[17] Healthwatch England . Personal communication. 2019.

