# Supplementary material for: Enabling ‘citizen voice’ in the English health and social care system: A national survey of the organizational structures, relationships and impacts of local Healthwatch in England
Source: Health Expect. 2020 Jul 7;23(5):1108–17. doi: 10.1111/hex.13086 (PMC7696119; doi:10.1111/hex.13086)
Supplement: Supplementary file 1 — Supplementary Material [file HEX-23-1108-s001.pdf]

Healthwatch national survey FINAL (copy)

Thank you for agreeing to take part in our survey.

The information you provide will remain confidential and we will anonymise all data. Please see more information about data use and protection below ('How will we use the information you provide in this survey?').

About the survey

The survey asks questions about:

- how your Healthwatch is structured
- how work is divided between employed staff and volunteers
- how your Healthwatch chooses its priorities
- what factors help or hinder your Healthwatch's ability to make a difference for your local community
- whether you would like to be further involved in our study.

Who should complete the survey?

The survey should be completed by the CEO or manager of a local Healthwatch.

- Where the local Healthwatch is run as part of a larger, non-Healthwatch organisation, the person with specific responsibility for Healthwatch operations and strategy should complete the survey (e.g. the local Healthwatch manager).
- Where two or more local Healthwatch services are run in a unified way by a single, over-arching Healthwatch team (e.g. Healthwatch X-shire and Healthwatch Y-City are run by an organisation called ‘Healthwatch X-shire and Y-City’), we suggest that the CEO or manager for the combined Healthwatch complete only ONE survey.

About us and our study

We are researchers from King’s College London and the University of Cambridge. We’ve received funding from the National Institute for Health Research to explore and enhance the local operation and impact of Healthwatch in England (HS&DR - 17/05/110).

In co-operation with local Healthwatch, we will provide a detailed understanding of Healthwatch work and relationships. The study will generate practical recommendations on how to ensure that all stakeholders get the most out of patient and public voice in health and social care commissioning and service provision.

How will we use the information you provide in this survey?

We will analyse the responses and also use the findings as part of the process of choosing four potential study-sites for in-depth, on-the-ground research which will form the second phase of the project.

We will ensure confidentiality of your responses and comply with GDPR and other data protection legislation. No-one outside the study team will have access to this data and it will be held securely on King’s College London servers. We will keep this data for seven years.

If you have any questions, please contact the study team at healthwatchstudy@kcl.ac.uk

We would be grateful if you could complete the survey by **5pm Friday 11th January 2019**.

You will be able to save your progress and complete the survey over several sittings. Please click on ‘**Finish later**’ at the bottom of each page to do this.

Please remember to click '**FINISH**' when you reach the end of the survey.

1. On behalf of which local Healthwatch are you responding?

About you

2. What position do you hold at your local Healthwatch?

3. How long have you been in this post at this Healthwatch? Please give your answer in **number of years**.

Please enter a whole number (integer).

About your Healthwatch

4. Please select the statement which best describes your Healthwatch.

- ○ We are an independent social enterprise that only does Healthwatch work
- ○ We are hosted by another organisation

5. Please select all the statements which accurately describe your Healthwatch's formal associations and structure.

- ☐ We are funded by one local authority
- ☐ We are jointly commissioned by two or more local authorities
- ☐ We have formed a consortium with one or more other Healthwatch
- ☐ Our Board of Directors oversees two or more Healthwatch
- ☐ We have a 'host' organisation which holds more than one Healthwatch contract
- ☐ We subcontract all or part of our Healthwatch functions to other suppliers
- ☐ None of the above

5.a. If you selected 'None of the above', please describe your formal structure and associations:

6. If you would like to describe your structure and associations in more detail, please do so here. *Optional*

Funding

7. How is your Healthwatch funded by its local authority for its core Healthwatch functions?

- ○ Contract
- ○ Grant
- ○ Other

7.a. If you selected Other, please specify:

8. Would you like your local authority to change the mechanisms through which your Healthwatch is funded?

- ○ Yes
- ○ No
- ○ I don't know

9. How would you like your local authority to change the mechanism through which you are funded? Please select all that apply.

- ☐ Move from grant to contract
- ☐ Move from contract to grant
- ☐ Lengthen the funding period
- ☐ Shorten the funding period
- ☐ Co-commission services with another local authority
- ☐ I don't know
- ☐ Other

9.a. If you selected Other, please specify:

External funding

10. Has your Healthwatch received sources of funding in your capacity as a Healthwatch provider that are external to the local authority Healthwatch contract?

- Yes
- No

11. Please indicate the sources of this external funding. Please choose all that apply.

- ☐ A local authority covered by this Healthwatch
- ☐ Another local authority
- ☐ Jointly by more than one local authority
- ☐ One CCG
- ☐ Jointly by more than one CCG
- ☐ Sustainability and Transformation Partnership (STP)
- ☐ Commissioning Support Unit
- ☐ NHS provider organisation
- ☐ Non-NHS provider of healthcare services (i.e. private healthcare sector)
- ☐ Third sector organisation
- ☐ Private social care sector
- ☐ Universities/Higher Education sector
- ☐ Big Lottery Fund
- ☐ Private sector charitable funding (e.g. private trusts or foundations)
- ☐ Earned income (e.g. from consultancy fees)
- ☐ Other

11.a. If you selected Other, please specify:

12. What are the service(s) for which external funding is provided? Please choose all that apply.

- ☐ NHS Complaints Advocacy Service
- ☐ Development of patient/public engagement initiatives
- ☐ Research on patient or service user experience

- [ ] Facilitation of focus groups
- [ ] Distribution of materials or information
- [ ] Evaluation of service or initiative
- [ ] Training and education
- [ ] None - external funding was philanthropic or not dependent on providing an extra service
- [ ] Other

12.a. If you selected Other, please specify:

Healthwatch as funder

13. Does your Healthwatch award funding (e.g. grant, contract etc) to other organisations?

- Yes
- No

13.a. If you selected Yes, please provide details:

The daily work of your Healthwatch

All Healthwatch in England rely on the work of employed staff and volunteers. Please estimate what proportion of the following activities are conducted by staff and volunteers in your Healthwatch.

14. Enter and View visits

15. Carrying out outreach and engagement activities

16. Administrative and clerical work

17. Research and report writing

18. Communications and social media

19. Attending meetings as your Healthwatch's representative (e.g. at Health and Wellbeing Boards etc.)

20. Taking part in local consultations about health and social care

21. Information and signposting

22. Contracted advocacy service for social care

23. Mystery shopping

Priorities and guidance for action

24. Most Healthwatch identify priority areas to help focus their work. How did your Healthwatch choose its priorities this year? Please select all that apply.

- ☐ We reviewed our signposting/information database
- ☐ We reviewed patient or service-user feedback
- ☐ We engaged with local people through bespoke events or surveys
- ☐ We adopted the priorities of the local health and social care system
- ☐ We were guided by an impending CQC inspection or published CQC report
- ☐ We responded to local health charity or voluntary sector concerns
- ☐ We used a weighting tool for prioritising issues which was approved by our Board
- ☐ We aligned with our host organisation's priorities
- ☐ We considered local commissioning intentions (e.g. planned recommissioning of a service)
- ☐ We addressed findings from national patient surveys (CQC and NHS England)
- ☐ We addressed 'hot topics' in the local or national news
- ☐ We negotiated priorities with neighbouring local Healthwatch
- ☐ Other
- ☐ Not applicable

24.a. If you selected Other, please specify:

25. Do you use any of the following to structure the activities of your Healthwatch? Please select all that apply.

- ☐ National Institute for Health and Care Excellence (NICE) Guidelines
- ☐ NHS Constitution
- ☐ CQC Inspection Frameworks
- ☐ Quality and Outcomes Framework for GPs
- ☐ Local authority Service Level Agreement and monitoring frameworks
- ☐ CCG Service Level Agreement and monitoring frameworks
- ☐ NHS England Bite-Size guide to Insight and/or Participation
- ☐ NIHR INVOLVE guidance on patient/public involvement in research
- ☐ Gunning Principles on public consultations
- ☐ Human Rights Act 1998
- ☐ Equality Act 2010
- ☐ None of the above
- ☐ Other

25.a. If you selected Other, please specify:

Relationships

How many of the following do you engage with in your work?

26. CCGs

26.a. Are they all located within the boundaries of your local authority?

- Yes
- No
- Not applicable

27. Hospital trusts

27.a. Are they all located within the boundaries of your local authority?

- Yes
- No
- Not applicable

28. Mental health trusts

28.a. Are they all located within the boundaries of your local authority?

- Yes
- No
- Not applicable

29. Community health trusts

29.a. Are they all located within the boundaries of your local authority?

- Yes
- No
- Not applicable

Relationships (continued)

How many of the following do you engage with in your work?

30. GP surgeries

- 1-10
- 11-20
- 21-30
- 31-40
- More than 40

30.a. Are they all located within the boundaries of your local authority?

- Yes
- No
- Not applicable

31. Care homes

- 0
- 1-10
- 11-20
- 21-30
- 31-40
- 41-50
- More than 50

31.a. Are they all located within the boundaries of your local authority?

- ○ Yes
- ○ No
- ○ Not applicable

32. Specialist service (e.g. St Andrews specialist mental health centre, congenital heart defect centre etc.)

- ● One or more
- ○ None
- ○ I don't know

32.a. If you selected 'one or more', please give details of the specialist service. *Optional*

32.b. Are the specialist services all located within the boundaries of your local authority?

- ○ Yes
- ○ No
- ○ Not applicable

32.b.i. If you would like to provide more detail, please do so here. *Optional*

The wider local health and social care landscape

33. How would you describe the overall quality of co-operation among key health and social care stakeholders in your local area?

- Excellent
- Good
- Neither good nor bad
- Limited
- Poor
- I don't know

34. In a major change to the health care landscape, Sustainability and Transformation Partnerships (STPs)/Integrated Care Systems (ICS) are being developed throughout England. To what extent has your Healthwatch been involved in the development of STPs/ICSs?

- An high level of involvement
- A good level of involvement
- Some involvement
- Not much involvement
- No involvement
- Not applicable

35. How has your level of involvement in STP/ICS work changed compared to this time last year?

- Involvement has increased
- Involvement has decreased
- Involvement has stayed the same
- I don't know

Impact

36. In the past 12 months, has your Healthwatch engaged in activites which produced any of the following types of impact?

- ☐ Produced changes to local contract specifications for a health and social care service
- ☐ Produced changes to the way health and social care services are monitored by commissioners
- ☐ Influenced new commissions or commissioning intentions
- ☐ Influenced the outcome of CQC inspections
- ☐ Involvement in quality improvement projects
- ☐ Improved access to care and treatment for members of our local community
- ☐ Increased levels of participation in co-production of people who use a service
- ☐ Prompted a Local Authority Overview and Scrutiny Committee to investigate an issue we identified
- ☐ Promoted issues which were adopted into a strategy (locally, regionally or nationally)
- ☐ Escalated an issue to Healthwatch England which was later actioned
- ☐ Produced changes to CQC inspection frameworks
- ☐ Produced changes to NICE guidelines
- ☐ Influenced changes in national policy or specialist commissioning

Think about a key piece of work you have done in the past 3 years which you regard as successful.

37. What was the piece of work about?

38. What was the key impact you achieved?

38.a. If you selected Other, please specify:

39. How long did it take to achieve this impact?

40. How was the impact delivered (e.g. research presenting evidence, publicity activity etc.)?

41. Which of the following local stakeholders did you involve to achieve this impact. Please select the three most important.

Please select between 1 and 3 answers.

- [ ] Health and Wellbeing Board members
- [ ] Local authority Overview and Scrutiny Committee
- [ ] Local authority commissioners of Healthwatch
- [ ] Local authority public health department
- [ ] Adult Safeguarding board
- [ ] Local councillors
- [ ] Local MPs
- [ ] CCG board and staff
- [ ] Local STP/ICS board
- [ ] Local CQC inspectors
- [ ] Staff of neighbouring Healthwatch
- [ ] Lay members of local healthcare bodies (e.g. CCG, Governors of Trusts etc.)
- [ ] Local patient or condition-specific groups
- [ ] GP surgeries
- [ ] Patient experience leads at hospital trusts
- [ ] Community voluntary sector organisations
- [ ] Social care providers
- [ ] Media
- [ ] Other

41.a. If you selected Other, please specify:

Barriers to impact

Now think about a key piece of work you carried out in the past 3 years which failed to make the impact you wanted.

42. What was the piece of work about?

43. What was the impact you wanted to achieve?

- Produce changes to local contract specifications for a health and social care service
- Produce changes to the way health and social care services are monitored by commissioners
- Influence new commissions or commissioning intentions
- Influence the outcome of CQC inspections
- Involvement in quality improvement projects
- Improve access to care and treatment for members of our local community
- Increase levels of participation in co-production of people who use a service
- Prompt a Local Authority Overview and Scrutiny Committee to investigate an issue we identified
- Promote issues which were adopted into a strategy (locally, regionally or nationally)
- Escalate an issue to Healthwatch England for action
- Produce changes to CQC inspection frameworks
- Produce changes to NICE guidelines
- Influence changes in national policy or specialist commissioning
- Other

43.a. If you selected Other, please specify:

44. Please briefly describe the barriers to impact you experienced.

Further involvement in the study

Thank you for taking the time to complete our survey.

There are two further ways you can join us in our project and we'd like you to indicate your interest below. Please note that expressing willingness to participate does not commit you at this stage.

You can find more information about our study here.

45. We would like to work with four local Healthwatch as study-sites for more in-depth research. Becoming a study-site is a fantastic opportunity to reflect on what you do and how to get the best out of your relationship with other organisations. **Would your Healthwatch be interested in being a study site?**

- Yes
- No
- I'm not sure

46. We are also looking for fifteen other Healthwatch to join our Healthwatch Involvement Panel. This Panel will help the study team analyse emerging data at meetings in London and enable us all to get a broader perspective on how Healthwatch works. Members of the Panel will be both employed staff and volunteers, and travel and other costs will be covered. Taking part as a Panel member will give your Healthwatch a chance to shape our research as it unfolds and learn about how other members of the Healthwatch network work. **Would someone in your Healthwatch like to participate in the Healthwatch Involvement Panel?**

- Yes
- No
- I'm not sure

Comments

47. If you have any comments about the survey or study, please share them with us here. *Optional*

Please press 'Finish' now.

Final page

Thank you for completing our survey.

The study is funded by the National Institute for Health Research's Health Services and Delivery Research Programme (Study ID 17/05/110). The Principal Investigator is Professor Glenn Robert, Florence Nightingale Faculty of Nursing, Midwifery and Palliative Care, James Clerk Maxwell Building, 57 Waterloo Road, London SE1 8WA.

To ensure our research meets ethical standards, the study is registered on the King's College London Minimal Risk Register (reference number MRA-18/19-8494).

If you have any questions or comments, please contact the study team at healthwatchstudy@kcl.ac.uk

---

Key for selection options

14 - Enter and View visits
- Wholly by employed staff
- Mostly by employed staff with some volunteer contribution
- Equally by employed staff and volunteers
- Mostly by volunteers with some employed staff contribution
- Wholly by volunteers
- Not applicable

15 - Carrying out outreach and engagement activities
- Wholly by employed staff
- Mostly by employed staff with some volunteer contribution
- Equally by employed staff and volunteers
- Mostly by volunteers with some employed staff contribution
- Wholly by volunteers
- Not applicable

16 - Administrative and clerical work
- Wholly by employed staff
- Mostly by employed staff with some volunteer contribution
- Equally by employed staff and volunteers

Mostly by volunteers with some employed staff contribution
Wholly by volunteers
Not applicable

17 - Research and report writing

Wholly by employed staff
Mostly by employed staff with some volunteer contribution
Equally by employed staff and volunteers
Mostly by volunteers with some employed staff contribution
Wholly by volunteers
Not applicable

18 - Communications and social media

Wholly by employed staff
Mostly by employed staff with some volunteer contribution
Equally by employed staff and volunteers
Mostly by volunteers with some employed staff contribution
Wholly by volunteers
Not applicable

19 - Attending meetings as your Healthwatch's representative (e.g. at Health and Wellbeing Boards etc.)

Wholly by employed staff
Mostly by employed staff with some volunteer contribution
Equally by employed staff and volunteers
Mostly by volunteers with some employed staff contribution
Wholly by volunteers
Not applicable

20 - Taking part in local consultations about health and social care

Wholly by employed staff
Mostly by employed staff with some volunteer contribution
Equally by employed staff and volunteers
Mostly by volunteers with some employed staff contribution
Wholly by volunteers
Not applicable

21 - Information and signposting

Wholly by employed staff
Mostly by employed staff with some volunteer contribution
Equally by employed staff and volunteers
Mostly by volunteers with some employed staff contribution
Wholly by volunteers
Not applicable

22 - Contracted advocacy service for social care
Wholly by employed staff
Mostly by employed staff with some volunteer contribution
Equally by employed staff and volunteers
Mostly by volunteers with some employed staff contribution
Wholly by volunteers
Not applicable

23 - Mystery shopping
Wholly by employed staff
Mostly by employed staff with some volunteer contribution
Equally by employed staff and volunteers
Mostly by volunteers with some employed staff contribution
Wholly by volunteers
Not applicable

26 - CCGs
0
1
2
3
4
5
More than 5
I don't know

27 - Hospital trusts
0
1
2
3

4
5
More than 5
I don't know

28 - Mental health trusts

0
1
2
3
4
5
More than 5
I don't know

29 - Community health trusts

0
1
2
3
4
5
More than 5
I don't know

38 - What was the key impact you achieved?

Produced changes to local contract specifications for a health and social care service
Produced changes to the way health and social care services are monitored by commissioners
Influenced new commissions or commissioning intentions
Influenced the outcome of CQC inspections
Contributed to projects that have improved the quality of care
Improved access to care and treatment for members of our local community
Increased levels of participation in co-production of people who use a service
Prompted a Local Authority Overview and Scrutiny Committee to investigate an issue we identified
Promoted issues which were adopted into a strategy (locally, regionally or nationally)

Escalated an issue to Healthwatch England which was later actioned
Produced changes to CQC inspection frameworks
Produced changes to NICE guidelines
Influenced changes in national policy or specialist commissioning
Other
